# Supplementary material for: Are carbon emissions trading and green financial instruments synergistic? -Comprehensive quantitative research based on content analysis
Source: PLoS One. 2024 Mar 7;19(3):e0298601. doi: 10.1371/journal.pone.0298601 (PMC10919601; doi:10.1371/journal.pone.0298601)
Supplement: S1 Data — (ZIP) [file pone.0298601.s001.zip › Data/地级市Z_F工作报告.zip!/╦─┤¿/─┌╜¡╩╨/└┤╫╘╣1⁄2╓┌║┼í╛┬φ┐╦╩2╛▌═°í┐_2016.docx]

各位代表：

　　我代表市人民政府，向大会报告工作，请予审查，并请市政协委员提出意见。

一、2015年及“十二五”工作回顾

　　2015年，面对经济下行压力持续加大的严峻形势，在省委、省政府和市委的坚强领导下，在市人大、市政协的监督支持下，市政府紧紧依靠全市人民，依法履行政府职能，主动适应经济发展新常态，大力实施“三大发展战略”，始终保持专注发展、转型发展定力，统筹抓好稳增长、促改革、调结构、惠民生、防风险各项工作，发展质量、发展效益、发展后劲明显增强，各项事业全面进步，社会大局和谐稳定，人民群众安居乐业。全市实现地区生产总值1198.6亿元，增长8.0%；地方一般公共预算收入50.3亿元，增长10.9%；农村居民人均可支配收入11428元，增长9.7%；城镇居民人均可支配收入25787元，增长8.1%，较好完成了六届人大五次会议确定的各项目标任务。

　　（一）稳增长，经济运行保持平稳

　　工业企稳回升。建立和落实“五项制度”（1），强化指标运行监测，推动工业经济止滑企稳、逐步回升。狠抓帮扶解困，安排各类工业发展资金1.3亿元，解决难题149个，帮助19户停产半停产规模工业企业恢复生产。重视大企业大集团扶持和中小企业培育，新增规模以上工业企业35户、“新三板” （2）上市企业3户。引导企业加大投入，完成工业技改投资228.7亿元，增长13.2%。帮助企业开拓市场，52个工业大类产品中，32个产品产量同比增长，工业产销率达98.8%。七大重点园区（3）完成主营业务收入1600亿元，新增承载面积3平方公里，新入驻单项投资5000万元以上企业（项目）28个。全市实现工业总产值1657亿元，增长5.4%；规模工业增加值增长8.1%；工业入库税金达13亿元。

　　投资高位增长。出台推进重点项目16条措施（4）和三级调度制度，每季度集中开工一批项目。256个市级以上重点项目完成投资384.7亿元，其中11个省级重点项目完成投资16亿元。产业投资完成201.6亿元，中铁建西南工业园、梓橦宫药业改扩建等项目加快推进，天津浩物汽车曲轴项目竣工投产。市政基础设施投资完成40.9亿元，建成北环线三四段、大千路北延线、清溪路等9条城市道路，资中县城南新区综合体、威远县婆城公园、隆昌县人民大桥落成。交通基础设施投资完成43.4亿元，成渝客专、自隆高速、内威荣高速建成通车，省道426田家至高桥段完成一级路改造，内江城市过境高速、内江综合客运中心开工建设，花园滩大桥项目建设顺利推进。信息基础设施投资完成12.2亿元，4G无线通信网络实现城市、乡镇全覆盖。全社会固定资产投资完成803.8亿元，增长15.2%，增速居全省第四位。

　　消费稳定增长。着力活跃城乡市场，加快培育汽车、信息等消费热点，成功举办迎春购物节、大千龙舟经贸文化节、大千网交会等展销活动，内江万达广场、红星美凯龙等商场相继开业，全市实现社会消费品零售总额408.6亿元，增长12.4%。大力推进棚改货币化安置，市本级棚改项目货币化安置率100%，我市的基本做法在全国、全省交流推广；落实楼市新政，商品房销售面积增长59.2%，增速居全省第一位。

　　（二）调结构，产业升级步伐加快

　　新型工业化推进加力。全市园区产业集中度提高3.5个百分点，创建国家高新区工作顺利推进，市中区工业集中发展区被评为全省机械制造品牌示范区，内江经济技术开发区被纳入全省首批信息安全产业示范园区。节能环保、信息安全、页岩气等高端成长型产业加速培育，高新技术产业、战略性新兴产业产值占规模工业总产值的比重达27.4%、18.8%，分别提高11.2个、3个百分点。设立2000万元市级科技孵化和成果转化专项资金，全市建成科技企业孵化器9个（其中省级5个），实施市级以上重大科技成果转化项目51项。新建院士（专家）工作站2个、省级工程技术研究中心1个、省级企业技术中心2个、市级企业技术中心22个，新获批国家高新技术企业23户。新增专利申请超过1000件，新增中国驰名商标2枚、四川省著名商标5枚。

　　现代服务业发展加速。川南电商中心项目加快建设，农融网、中国禽苗交易网等一批电商平台建成投用，市中区荣登“2015四川电商区域竞争力”榜单，资中县“国家级电子商务进农村综合示范县”建设成效明显，全市电子商务交易额实现522亿元、居全省第三位，增长44%。按照“把城市变成景区、把资源变成产品、把旅客变成游客”的思路，加快实施旅游发展“一三八”计划（5），古宇湖AAAA级景区成功创建，石板河景区一期工程对外开放，小青龙河、塔山公园景观绿道等项目加快推进，实现旅游总收入170.5亿元，增长24.5%。新引进域外金融机构3户。银行业金融机构本外币各项存款余额1204.5亿元,比年初增加67.3亿元，增长6.0%；贷款余额688.8亿元，比年初增加78.6亿元，增长12.9%。

　　农业现代化进程加快。省级现代农业重点县、畜牧业重点县、林业重点县通过验收，成功创建国家农业科技园区，威远县、资中县分别获评全省“三农”工作、农民增收工作先进县，镇西食品工业园区被命名为“全国农业产业化示范基地”。改造提升现代农业产业基地5.8万亩，发展林产业基地12.3万亩，建成现代农业万亩亿元示范区11个，新增规模以上龙头企业12户、农民合作组织122个、家庭农场294个、种养大户419户，生猪、家禽适度规模养殖面分别达71%、56.6%。持续推进兴水惠民“五年行动计划”，联合水库加快建设。强化耕地保护，耕地实际保有量、基本农田保护面积分别达27.3万公顷、21.4万公顷。农业增加值增长3.9%。

　　（三）促改革，发展活力不断释放

　　各项改革有序实施。新推出17项专项改革方案，全力推进38项重点改革事项。扎实推进简政放权，在全省率先公开230个市级行政审批项目，项目审批提速87.1%；打造阳光交易平台，公共资源交易实现“九个统一”（6）；深入实施“三证合一、一照一码”登记制度（7）改革，新登记各类市场主体1.7万个，增长28.4%。农村产权“五权同确”（8）全面完成，全国第二批农村改革试验区建设等改革试点扎实推进，“中国改革（2015）年会暨农村产权制度改革高层研讨会”在内江举办。全市农信社改制组建农商行工作加快推进。深化文化体制改革，传统媒体与新兴媒体加快融合，户外多媒体阅报系统获评“王选新闻科学技术奖”（9）。供销社综合改革、不动产登记、国资管理体制、事业单位分类等改革稳步实施。

　　开放合作成效明显。成功举办川南电商博览会、第八届中国国际中小企业交易会、川商回家发展恳谈会等系列开放活动，全市实际到位市外资金477.5亿元，增长15.1%，其中制造业项目到位资金占68.8%。到位市外资金形成固定资产投资239.2亿元，固定资产投资转化率（10）达50.1%。实现外贸进出口总额1.4亿美元，实际利用外资0.7亿美元。大力实施返乡创业“回家工程”，新创办经济实体174个，新增就业1.6万人。

　　（四）惠民生，人民生活持续改善

　　民生工程扎实推进。“十项民生工程”、20件民生大事全面完成，民生支出达135.4亿元，占一般公共预算支出的70.4%。制定工资集体协商工作等级评价体系地方标准，新增城镇就业4.9万人，城镇登记失业率控制在3.9%。社会保障体系不断完善，五大保险覆盖人数达333.1万人次，城乡居民基本医疗保险补助上调至380元/人·年，城乡居民低保标准分别提高至4800元/人·年、2460元/人·年，新农合居民参合率达99.8%。完成扶贫投资2.3亿元，帮助4万农村贫困人口实现脱贫。对符合条件的失独家庭父母，给予6000元/人·年的扶助金。新建区域性养老服务中心4个、社区日间照料中心87个，新增、改造养老床位4335张，为12.5万名困难家庭失能老人和80周岁以上老人提供居家养老服务。开工建设保障性住房934套,向农民工定向提供公租房441套,实施农村危房改造3765户。改造133个老旧居民小区供配电设施，解决12.3万人用电安全问题。有效解决26.4万农村人口、2.3万学校师生安全饮水问题，持续开展农村水井水质监测。新建农村户用沼气池6153口，建成公路安全护栏392.7公里。深入开展城区交通治堵，邱家嘴立交桥主体工程建成通车，沱江大桥加宽改造工程开工建设。深化中心城区市容市貌综合整治，拆除市区及城郊结合部违章搭建14.7万平方米，市容秩序和环境卫生管理工作迈入常态化，城市形象不断提升。

　　社会事业全面进步。学前教育三年毛入学率达87.2%，威远县、隆昌县义务教育均衡发展通过省上验收，藏区“9+3”中职免费教育毕业生100%就业，4.2万名农村义务教育在校学生享受了免费营养午餐，内江师院新校区、内江六中高新校区开工建设，桐梓坝小学整体迁建主体工程完工。继续实施分级诊疗制度，县域内就诊率达90.3%，市一医院、市二医院、市中医院新区项目加快建设，四川健康职业学院筹建工作顺利推进。低生育水平保持稳定，人口自然增长率4.5‰。市文化中心开工建设，开展百姓大舞台、公益大讲堂、书画大展场等群众文化活动600余场次。承办省级以上体育赛事9次，开展“周末快乐体育”活动350余场次。

　　生态环境得到改善。总量减排、污染防治任务全面完成。淘汰黄标车和老旧车4803辆，中心城区空气质量达到和好于Ⅱ级以上天数325天,可吸入颗粒物PM10年均浓度值为83微克/立方米，比省政府下达目标低13微克/立方米。全市县级以上集中式饮用水水源地水质达标率100%，中心城区污水集中处理率89%、生活垃圾无害化处理率98.6%。加快创建省级森林城市，成功创建生态乡镇5个、生态村20个，森林覆盖率达33.1%。

　　（五）重法治，社会秩序和谐稳定

　　依法治理能力提升。建成省级依法行政示范单位13个，调解处置各类矛盾纠纷3万余件。开展“六五”普法和“法律七进”活动，办理法律援助案件1782件。深化“平安内江”建设，加强社区矫正工作，网格化服务实现全覆盖，公众安全感满意率达95.4%。应急处置能力不断提升，有效防范暴恐案件和个人极端事件，各类突发事件得到及时妥善处置。

　　安全生产成效显著。夯实基层基础，建成全国安全社区1个、全省安全社区17个，我市被表彰为“全国安全生产月”活动先进单位。严格落实安全生产法规制度，按照“全覆盖、零容忍、严执法、重实效”的要求，依法开展常态化安全生产执法检查和隐患排查治理，安全生产“四项指标”全面下降，伤亡事故起数、受伤人数、死亡人数、直接经济损失分别下降19%、24%、15%、44%，未发生重大安全生产事故。加强食品药品监管，未发生重大食品药品安全事件。

　　各项工作协同推进。国防动员、人民防空、民兵预备役建设和双拥优抚等工作不断加强，军政军民关系更加巩固。机构编制、广播电视、防灾减灾、检验检疫、统计、调查、气象、水文、无线电管理、保密、档案、史志、外事侨务、民族宗教等工作迈出新步伐，红十字、慈善、老龄、妇女儿童、残疾人等事业取得新成绩。

　　（六）转作风，自身建设不断加强

　　扎实开展“三严三实”专题教育，严格执行改进工作作风各项规定，全市“三公”经费下降18.5%。自觉接受人大政协监督，办理人大代表议案2件、建议110件，满意率100%；办理政协提案284件，满意率99.7%。广泛听取民主党派、工商联、无党派人士、人民团体的意见，积极支持工会、共青团、妇联等群团组织开展工作。坚持政府常务会会前学法，完善法律顾问制度，依法决策、科学决策、民主决策水平有力提升。规范行政处罚裁量权，强化行政复议工作。深化政务公开，市、县行政权力公开运行平台实现全覆盖，清理并公开市级行政权力6461项。完成市级政府投资工程决算审计项目33个，审减资金4652万元。坚持“一岗双责”，严格落实党风廉政建设责任制，查处一批群众反映强烈的突出问题，政纪立案139件，政纪处分128人。

　　各位代表，2015年主要任务的顺利完成，标志着“十二五”规划目标如期实现。回顾“十二五”，我们高兴地看到：

　　这五年，综合实力明显增强。经济总量迈上1000亿元台阶，2015年主要经济指标与2010年相比：GDP增加508亿元，年均增长11.2%；人均GDP增加14059元，年均增长11.7%；地方一般公共预算收入增加近30亿元，年均增长19.8%；一般公共预算支出增加近100亿元，年均增长15.8%；全社会固定资产投资增加452亿元，年均增长18%;社会消费品零售总额增加206亿元，年均增长14.7%。市中区被纳入全国第二批农村改革试验区，东兴区、资中县被评为全省县域经济发展先进县（区），隆昌县被评为全国科技进步先进县、全省首批工业强县示范县，威远县被评为全省丘陵地区先进县，底部基础进一步夯实。

　　这五年，发展方式逐步转变。地方一般公共预算收入占GDP比重提高1个百分点，税收收入占地方一般公共预算收入比重提高6.6个百分点。集约节约用地水平大幅提升，单位GDP建设用地指标下降32.8%。淘汰落后企业160户，关闭小煤矿34户，淘汰水泥370万吨、平板玻璃206万重量箱。成功创建国家级经开区、省级高新区、省级新型工业化示范基地，建成科技企业孵化器10万平方米。成功纳入国家老工业基地调整改造规划、信息惠民国家试点城市（11）、“宽带中国”示范城市（12）。

　　这五年，城乡面貌显著改善。中心城区新增建成区面积31.8平方公里，三县县城建成区面积均超过20平方公里，城镇化率由2010年的39.6%，提升到2015年的45.6%。交通基础设施建设投资总量是“十一五”的3.4倍，县县通高速，村村通油路（水泥路），公路总里程超过1万公里，公路网密度居全省第二位。建成新农村综合体6个、幸福美丽新村150个。

　　这五年，改革开放全面深化。农村产权制度改革深入推进，“内江实践”在全省推广。财政预算改革、医疗卫生体制改革、要素价格改革等取得初步成效，改革红利逐步释放。开放合作取得突破，引进世界500强企业投资项目22个、中国500强企业投资项目33个、5亿元以上项目250个。

　　这五年，生活水平不断提高。财政用于民生的支出达561亿元，是“十一五”的2.5倍。2015年，城镇居民人均可支配收入比2010年增加11034元，年均增长11.8%；农村居民人均可支配收入比2010年增加5430元，年均增长13.8%。“十二五”期间，新增城镇就业25.5万人。建成偿还历史欠账安置还房137万平方米。教育、卫生、文化、体育等社会事业蓬勃发展。

　　过去的五年极不平凡。“三期叠加”（13）影响不断加深、老工业城市长期积累的矛盾日益凸显。我们主动适应经济发展新常态，在探索中前行、在拼搏中奋进、在砥砺中跨越，取得的成绩令人欣喜，收获的启示弥足珍贵！五年的进步和变化，得益于省委、省政府的正确领导、深切关怀，得益于市委的科学谋划、精心指挥，得益于市人大、市政协的有力监督、倾力支持，得益于全市干部群众的忘我工作、锐意进取。在此，我谨代表市人民政府，向430万内江人民，向人大代表、政协委员、各民主党派、工商联、无党派人士、各人民团体，向驻内部队、武警官兵、政法干警、民兵预备役人员，向关心、支持和参与内江建设发展的各界朋友，表示衷心感谢并致以崇高敬意！

　　各位代表，五年来的实践探索，我们深刻体会到：务必坚持发展第一。发展是解决一切问题的关键，一定要保持专注发展的战略定力，不断推进经济社会全面发展。务必坚持创新驱动。创新是引领发展的第一动力，一定要同步培育新兴产业、提升传统产业，不断推进创新发展、转型发展。务必坚持改革牵引。改革是推动发展的“火车头”，一定要充分发挥改革的先导性作用，不断推进各项改革，正确处理政府和市场的关系，努力激发全社会创新、创业、创造潜能。务必坚持民生至上。人民是促进发展的根本力量，一定要坚持以人民为中心的思想，不断推进发展经济与改善民生相结合，最大程度增强人民群众的获得感。务必坚持实干为本。求真务实、以干为先，方能不辱使命，一定要坚持在实干中把握机遇、化解矛盾，不断提升发展质量、壮大发展规模。这些成功实践，我们将倍加珍惜、继续坚持，努力推动政府工作迈上新台阶。

　　同时，我们也清醒地认识到，内江“底子薄、不协调、欠发达”的基本市情没有根本改变，发展还面临不少问题。一是发展差距仍然较大。人均GDP仅为全省平均水平的87.1%、全国平均水平的65.3%；服务业增加值增速、城乡居民人均可支配收入增速、城镇化率，未实现2015年预期目标。二是产业结构仍然不优。服务业占比明显偏低，传统产业面临转型阵痛，新兴产业起步晚、规模小。三是投资后劲仍然乏力。大项目、好项目少，资源要素日益趋紧，民间投资呈下降之势。四是实体经济仍然困难。部分行业产能过剩，一些企业运转困难，不少企业成本上升、盈利减少、亏损增加。五是民生问题仍然较多。城乡基础设施建设总体滞后，环境问题比较突出，安全隐患不断出现，教育、医疗、养老等公共服务与群众期盼还有差距。六是行政效能仍然不高。少数政府机关工作人员存在“懒政、怠政”现象，依法行政的素质和能力不能很好适应形势发展要求，行政效率还需提升。在今后工作中，我们将视差距为机遇、变压力为动力，正视问题、知难而进、主动作为，努力推进经济社会持续健康发展。

二、“十三五”发展的主要考虑

　　“十三五”时期是全面建成小康社会的决胜阶段，也是我市适应经济发展新常态、加快转型发展的关键时期。根据市委关于“十三五”规划编制的建议，市政府组织编制了《内江市国民经济和社会发展第十三个五年规划纲要（草案）》，已印发大会审议。这里，我主要从四个方面作简要说明。

　　关于发展形势。今后五年，我们面临的国内外发展形势依然错综复杂。从国际看，发展环境有望总体保持和平稳定，但国际金融危机的深层次影响还在持续显现，世界经济在深度调整中曲折复苏。从国内看，我国仍处于可以大有作为的重要战略机遇期，经济长期向好的基本面没有改变，但经济增速、工业品价格、实体企业盈利、财政收入增幅下降，经济风险发生概率上升。从全省看，经济发展进入规模质量同步提升期、工业化城镇化加速期、多点多极发展整体跃升期、发展动力转换关键期、产业转型接续期、全面建成小康社会决胜期，既积蓄了重要动能和积极因素，又面临转型阵痛和严峻挑战。

　　经过多年艰苦努力，我市产业发展、基础设施建设等方面取得长足进步，发展基础和发展条件明显改善。国家实施“一带一路”（14）、长江经济带、成渝经济区等发展战略，四川成为全国全面改革创新试验区、推动川南经济区率先突破等机遇，有利于内江在更高层面配置资源、布局项目。同时，作为老工业城市，我市传统产业占比高的现状还未根本改变，新常态带来的影响可能会更广泛、更持久、更深远，必须更加积极稳妥地应对。

　　关于奋斗目标。着眼加快建设幸福美丽内江，与全国、全省同步全面建成小康社会，我们提出的预期目标是：“十三五”期间，全市 GDP年均增长7.5%；到“十三五”末，居民人均可支配收入超过2.5万元，年均增长7.5%；服务业增加值比重达27.2%，提高3个百分点；常住人口城镇化率达53%，提高7.5个百分点。

　　到2020年，我们将建成指标协调的全面小康，经济总量和居民收入同步提高，在2010年的基础上翻一番；就业持续扩大，物价更趋合理；绝对贫困彻底消除，贫困村全部“摘帽”，收入差距、城乡差距日益缩小；社会保障更加充分，社会风气更加文明，幸福指数不断提高。我们将建成和谐宜居的“双百”城市，中心城区面积突破100平方公里、常住人口突破100万人，新城展活力、旧城换新颜，成功打造“四方块”等一批“留住记忆、唤起乡愁”的城市名片，现代都市、现代产业、现代生活融合发展。建成国家卫生城市、信息惠民国家示范城市，构建起现代化城市管理长效机制。我们将建成特色鲜明的旅游城市，一批“城市变景区”项目建成投用，旅游发展“一三八”计划全面完成，内江成为成渝地区新兴休闲度假旅游目的地。我们将建成外通内畅的枢纽中心，内江城市过境高速公路、中心城区至三县快速通道、川南城际铁路、连乐铁路等全面建成，五个“30分钟内江” （15）和“县县通高铁”全面实现，力争纳入国家“公交都市”试点城市（16），成渝经济区枢纽地位更加巩固。我们将建成天蓝水净的美好家园，沱江饮用水源地水质常年达标；向家坝灌区北总干渠向内江引水工程建成投用，让中心城区和隆昌城区居民饮用金沙江水；资中城北水资源——河库联网工程建成使用，威远河、隆昌河等主要流域水质明显改善。中心城区和三县县城空气质量优良率持续提升。建成省级森林城市，全市森林覆盖率达35%。

　　关于主攻方向。打好“三大攻坚战”，弥补发展短板，破解制约全面建成小康社会的难题。打好产业发展攻坚战，把“工业强市、产业兴市”作为内江科学发展、加快发展的首要任务，按照“扩总量、活存量、抓增量、优质量、聚力量”的要求，实施“七大行动”（17），打造“一中心四基地”（18），推进“三个转变”，推动产业发展、转型升级。打好城乡基础设施建设攻坚战，统筹规划、适度超前，加快建设交通、市政、水利、环保、能源、信息等基础设施，不断改善城乡发展条件。打好脱贫攻坚战，把脱贫攻坚作为最大民生抓紧抓实，解决好“扶持谁、谁来扶、怎么扶”的问题，在精准施策、精准帮扶、精准落地上下功夫、见实效。

　　关于发展路径。坚持创新、协调、绿色、开放、共享五大发展理念，走出一条符合内江实际的发展道路。坚持创新发展，突出理念创新、制度创新、科技创新，推进“大众创业、万众创新”，加快发展高新技术和战略性新兴产业，厚植经济增长新优势。坚持协调发展，落实主体功能区规划，推进城乡统筹、三产融合，支持符合条件的县撤县设市，增强县域经济发展活力，提升县域经济发展水平。坚持绿色发展，大力改善环境质量，构建生态文明新家园。坚持开放发展，提升招商引资水平，主动融入成渝、配套成渝，错位发展、借力发展，在更高层次、更宽领域汇聚资源要素。坚持共享发展，努力提供更优质的教育、更稳定的工作、更可靠的社会保障、更完善的医疗服务、更舒适的居住条件，让全市人民共享甜蜜生活。

　　三、2016年工作安排

　　2016年是实施“十三五”规划和决胜全面小康的开局之年。今年政府工作的总体要求是：全面贯彻党的十八大和十八届三中、四中、五中全会精神，以邓小平理论、“三个代表”重要思想、科学发展观为指导，深入贯彻习近平总书记系列重要讲话精神，按照“五位一体”总体布局和“四个全面”战略布局，牢固树立和贯彻落实“五大发展理念”，主动适应把握引领经济发展新常态，围绕省委十届六次、七次全会和市委六届十次全会部署，坚持稳中求进，坚持改革开放，坚持稳增长、调结构、惠民生、防风险，坚持工业强市、产业兴市，深入实施“三大发展战略”，全力加强供给侧结构性改革，坚决打好产业发展攻坚、城乡基础设施建设攻坚、脱贫攻坚“三大攻坚战”，加快培育发展新动能，着力构建共享发展新格局，努力为“十三五”发展开好局、起好步。

　　今年全市经济社会发展的主要预期目标建议：地区生产总值增长8.0%，其中第一产业增加值增长3.5%、第二产业增加值增长8.2%（其中规模工业增加值增长8.0%）、第三产业增加值增长10.0%；地方一般公共预算收入同口径增长8.3%；社会消费品零售总额增长12.0%；全社会固定资产投资增长12.0%；城镇居民人均可支配收入增长8.5%，农村居民人均可支配收入增长9.5%；居民消费价格涨幅控制在3.0%以内；城镇登记失业率控制在4.5%以内。

　　围绕目标，重点抓好以下七项工作。

　　（一）坚持工业强市、产业兴市，着力夯实发展支撑

　　推进工业提质增效。实施产业发展攻坚三年行动计划，构建以先进制造业为主导、三次产业协调发展的现代产业体系。做大规模，加快企业培育，新增规模工业企业20户以上；以七大重点园区为支撑，稳步提升全市工业园区投资强度和产出效率，产业集中度提高1个百分点以上，完成主营业务收入1700亿元。优化结构，加快老工业基地调整步伐，采用新技术、新工艺、新设备，改造提升传统产业，完成技改投资228亿元以上；推动医药化工向生物医药发展、冶金建材向新材料发展，增强机械汽配产业竞争力，壮大食品饮料产业，稳定电力能源产业；建设信息安全产业孵化园，在数据处理、物联网等领域形成有市场竞争力的产品，力争高新技术产业、战略性新兴产业产值占规模工业总产值比重分别提高3个、2.5个百分点。助企脱困，深化“五项制度”，定期分行业、分产业研究，强化金融、社保、税费、电力等支持，推动企业脱困发展。

　　促进服务业上档升级。加快建设谢家河省级商贸集聚区，逐步形成中心城区“一环两轴多片”（19）的商贸流通格局。大力发展电子商务，加快建设内江电商产业园，力争川南电商中心建成投用；实施电子商务进农村、进社区工程，保持电子商务全省领先地位。大力发展现代物流业，规范发展寄递物流，启动川南快递物流园项目，吸引国内外知名物流企业落户。继续推进旅游发展“一三八”计划，建成一批“城市变景区”项目，创建一批国家AAAA级旅游景区，新开工一批重大旅游项目，实现旅游总收入210亿元以上。大力发展会展经济；积极培育养老健康业；加快发展交通运输业。大力引进域外金融机构，创新金融产品、加大信贷投放，提升保险业发展水平，落实财政金融互动政策，加大金融支持实体经济力度。

　　转变农业发展方式。注重发展特色效益农业，推进5个万亩以上特色产业示范片建设，新建高标准农田8万亩，新增粮食产量1万吨。支持新型农业经营主体多元化发展，培育家庭农场50个、专业大户200户、农民合作社20个、农业产业化龙头企业5家。打造“内江猪”“资中血橙”“威远无花果”等品牌，提升农产品知名度。推进“互联网+农业”，积极开发农业多种功能，促进农村三次产业融合发展，大力发展休闲农业和乡村旅游，持续增加农民收入。做好第三次全国农业普查，全面摸清“三农”情况。

　　（二）坚持基础先行、协调发展，着力改善发展条件

　　加快大城市建设。坚持规划引领，完善城乡规划体系，编制完成中心城区绿地系统、地下管网、综合管廊等专项规划。加快内江新城建设，狠抓城南新区开发，大力实施高铁站前综合配套、邓家坝安置还房等61个新城核心区项目，加快甜都大道北延线、东兴大道北延线、石羊大道等道路建设。打造重要节点景观，加快实施甜蜜花园、清溪湿地南段工程，启动滨江北路综合整治工程（20）。完善配套设施，推进内江三水厂、谢家河再生水厂、内江市生活垃圾处理中心等项目建设，同步启动地下综合管廊建设。持续开展中心城区交通治堵工作，完成桐梓坝大桥南成渝铁路桥下道路拓宽项目，实施邱家嘴立交桥南延线、沱江大桥加宽改造、西林大桥南北端下穿隧道等项目，加快建设中心城区停车场，增设人行过街通道。加大危旧房棚户区改造力度，启动中心城区“四方块”改造提升工程，加快滨江东路片区、容器厂片区改造，完成老旧院落整治116个。强化城市管理，加速形成大城管格局，提升城市综合治理能力，拆除城区及城郊结合部违章搭建，整治城区违规占道经营和违规运输建筑垃圾，加强背街小巷、城中村、城郊结合部环境卫生治理，建成数字化城管系统，免费开放中心城区公厕。扎实推进创建国家卫生城市工作。

　　推动中小城镇发展。推进县域“多规合一”，把城乡规划实施纳入经济责任审计内容，确保实施过程规范有序。启动三县城市开发边界划定工作，完善县城绿地系统和历史文化保护规划，确保县城控规覆盖率在95%以上。引导县（区)积极创建全省县域经济50强，争创省级幸福美丽新村示范县。加快资中古城文化旅游产业示范园、苌弘大道北延线等项目建设，提升省级历史文化名城美誉度。加快隆昌县影视中心等配套设施建设，打造“全省最整洁县城”。加快威远县15平方公里县城新区建设，打造具有较强辐射带动能力的川南特色县城。加快白马、田家、银山、界市、连界等17个国、省重点镇建设，建成一批工业经济、商贸物流、现代农业、生态旅游强镇。抓好5个新村重点片、300个幸福美丽新村建设。完善转移人口与建设用地、建设资金、转移支付“三挂钩”机制；实施居住证制度，促进农村居民就近就地城镇化。全市城镇化率提升1.5个百分点，新增城镇建成区面积10平方公里。

　　加强基础设施建设。实施城乡基础设施建设攻坚三年行动计划，构建适度超前、功能配套的现代城乡基础设施体系。开工建设川南城际铁路、连乐铁路，加快建设内江城市过境高速公路、黄荆坝大桥、内江综合客运中心、内江公交枢纽中心，建成花园滩大桥，新（改）建农村公路100公里以上，力争完成交通基础设施建设投资20亿元。启动建设向家坝灌区北总干渠向内江引水工程，推进长征渠引水工程规划修编，加快建设联合水库，开工建设两河口水库，实施石盘滩、龙江水库灌区续建配套与节水改造。推进骨干电网建设和农村电网改造，加快建设内江新城线路迁改电力管廊、220KV资铁和110KV越溪输变电工程。继续推进“宽带中国”“智慧城市”建设，推行电信、电力、广电等杆路资源共建共享，光纤到户覆盖家庭突破90万户，实现全市行政村宽带全覆盖。建成内江大数据中心，完成信息惠民国家试点城市创建任务。

　　（三）坚持改革牵引、创新发展，着力增强发展动力

　　推进供给侧改革（21）。弄清全市产能现状，科学确定目标任务和实现路径，在争取国、省支持的基础上，多措并举化解过剩产能，积极稳妥处置“僵尸企业”。进一步减税降费，加强要素协调，强化技能培训，落实直购电、富余电量消纳等政策，加大贷款贴息、担保力度，狠抓企业股权挂牌和上市培育，推行差别化用地政策，不断降低企业生产性、经营性、外在性成本。支持农民工和农民进城购买商品房，引导房地产企业适当降低销售价格，加大棚改货币化安置力度，全市货币化安置率超过60%，助推商品房去库存。打击非法集资，积极处置不良贷款，落实存款保险制度（22），坚决守住不发生系统性、区域性金融风险的底线。加强政府性债务监测监管，规范双创债、小微债等举债行为，积极争取国家债务置换，保持债务风险在可控范围之内。

　　深化重点领域改革。深化农业农村改革，推进市中区的全国第二批农村改革试验区、东兴区的全省首批农村改革综合试验区建设等改革试点，依法推动土地经营权有序流转。推进简政放权，完善责任清单制度。深化事业单位改革，激发事业单位活力。加快公立医院改革，完善分级诊疗制度，力争县域内就诊率在90%以上。推进政务服务平台建设，加快建立全市统一规范的公共资源交易平台。深化国有企业改革，夯实市级国有平台。深化电力体制改革，探索组建区域性地方售电公司。深化价格改革，实施城区阶梯用水、用气价格制度。深化金融制度改革，规范发展农村资金互助组织，加快组建内江农商行、资中农商行，实现隆昌农商行挂牌运营。深化财税体制改革，全面落实“营改增”政策。加快不动产登记，确保年内颁发新证。完成公务用车制度改革，保障正常公务出行。

　　提升自主创新能力。坚持申报创建、创新创业、科技服务并重，加快推进白马新材料园等项目建设，确保成功创建国家高新区。加快建设创新平台，增强孵化器承载能力，新增入孵企业30户以上。推动建设企业研发机构，积极创建院士（专家）工作站、省级工程技术研究中心、企业技术中心，组织实施重大科技成果转化项目25项。加强知识产权保护，新增专利申请10%，新增国家地理标志保护产品1个，新培育四川省著名商标2枚，持续培育四川名牌产品29个。加快引进一批科研团队和高新技术人才。

　　（四）坚持投资拉动、开放发展，着力做大发展增量

　　努力扩大投资规模。扎实开展“项目年”活动，抓好235个市级重点项目和11个省级重点项目建设，确保完成市级重点项目投资200亿元以上、省级重点项目投资13.9亿元以上、全社会固定资产投资900亿元以上。健全项目全程服务机制，提高开工率、竣工率、投产率。推进城乡建设用地增减挂钩和工矿废弃地复垦利用试点，集约节约利用土地，确保近五年平均供地率高于65%。发挥政府性资金导向作用，积极推进PPP项目建设。把握国家和省投资政策取向，加强项目前期工作，提高项目成熟度，争取更多项目纳入国、省“盘子”。

　　继续深化开放合作。积极参与中外知名企业四川行、十六届西博会等投资贸易促进活动，引进一批产业带动强、税收贡献大、就业效果好的优质项目，全年到位市外资金525亿元以上，其中制造业项目资金占比不低于50%。深入实施返乡创业“回家工程”，引导农民工、农民企业家、在外内江知名人士返乡创业，确保引进返乡创业经济实体180个以上，实现投资总额120亿元以上。做大做强外贸龙头企业，引导企业拓展海外市场。加快川南经济区一体化发展，推动产业发展互补互促、基础设施互联互通、民生工程互惠互利，力争川南大数据中心、“川南一卡通”（23）落户内江。加强校地合作、校企合作，优选部分高校开展重点合作，发挥示范带动作用。

　　（五）坚持生态优先、绿色发展，着力建设美丽内江

　　强化法规贯彻。严格落实环境保护“党政同责、一岗双责”责任制，严格落实生态环境损害终身追究制和一票否决制。落实最严格的耕地保护、水资源管理制度，严守水资源、国土资源“三条红线” （24），建立国土空间开发保护制度，完成永久基本农田划定工作。严格执行环境影响评价和环保“三同时”（25）制度，开展排污权、水权交易探索研究。加强环境监测，依法公布各类环境监测数据，主动接受社会监督。

　　夯实生态基础。加快内江市第二污水处理厂和白马、椑木、连界、银山等重点集镇生活污水处理设施建设。推进资中县“全国生态文明示范工程试点县”建设。扎实开展省级森林城市创建工作，营林造林8万亩，全市森林覆盖率达33.6%。推进传统制造业企业绿色改造，推行绿色建筑标准，推动节能环保产业发展。开展工业废气、废水、废物的综合治理和回收利用，鼓励节水、节材，倡导绿色低碳的生产方式、消费模式、生活习惯，提高清洁能源在城市能源消费中的比重。

　　加强环境治理。抓好环保部西南督查中心综合督查反馈问题整改，着力解决区域性、结构性环境突出问题。健全大气联防联控机制，加快淘汰黄标车、老旧车，强力整治扬尘污染、露天焚烧污染、油煤烟污染，确保可吸入颗粒物PM10年均浓度值完成省下达的控制目标。继续实施“河长制”，推进沱江内江段、威远河、隆昌河、大清流河等主要流域深度治理，加强黄河湖、濛溪河等城乡重点饮用水源地保护。加大土壤、重金属和固体废物污染防治力度，强化医疗废物等危险废弃物收集、贮存、运输、处理全过程监管。推进重点企业节能减排，确保主要污染物总量减排和万元GDP综合能耗完成省下达目标任务。加强农村面源污染治理，改善农村生态环境。开展对违法排污问题的专项检查，依法查处环境违法行为。

　　（六）坚持以人为本、共享发展，着力增进民生福祉

　　办好民生实事。继续实施“十项民生工程”，确保今年民生支出占一般公共预算支出比重不低于全省平均水平。建立市创新创业活动中心，加快创建省级创业型城市，新增城镇就业3.7万人。实施全面参保计划，执行被征地农民社保资金预存款制度。减免1.1万名家庭经济困难高中学生学费，为3.7万名农村义务教育在校学生提供免费营养午餐。新建区域性养老服务中心15个、城乡社区日间照料中心85个，新增、改造养老床位3150张，为11.8万名困难家庭的失能老人和80周岁以上的高龄老人提供居家养老服务。深入实施保障性安居工程，开工建设保障性住房和改造棚户区8461套，定向为农民工提供保障房300套以上。扎实做好农村危房改造。继续开展农村水井水质监测，大力整改不合格水井，新增农村供水受益人口3.6万人。

　　狠抓脱贫攻坚。开展“四个一批”（26）脱贫攻坚行动，实施基础设施建设扶贫、产业扶贫等10个扶贫专项方案（27），做到目标、责任、资金、项目、措施、成效“六落实”。完善“七个一”帮扶机制（28），确保全市2.4万贫困人口脱贫、74个贫困村“摘帽”。积极推进农村电商扶贫，力争实现村级电商服务全覆盖。有序推进易地扶贫搬迁，安置贫困人口1794人。强化政策兜底扶贫，实行与新农合制度有效衔接的大病保险制度，完善农村最低生活保障等制度，实现建档立卡贫困户参加职业教育学生“雨露计划”（29）全覆盖。

　　发展社会事业。大力实施“社会事业十大惠民工程”（30），开工建设内江大剧院，加快建设内江师院新校区、内江六中高新校区、市文化中心、市体育中心，完成大千园改造提升、内江一医院新区主体工程；启动建设四川健康职业学院，开工建设市第二社会福利院整体迁建项目、市精神病康复院。支持内江职院、川南幼专健康发展。坚持计划生育基本国策，实施全面两孩政策。深入实施第二轮学前教育三年行动计划，新改扩建城乡公办幼儿园13所，新建义务教育标准化学校50所。抓好群众文化活动，实施文艺精品工程，科学保护文化遗产，启动“高清内江、智慧广电”工程建设。深入实施全民健身计划，完善社区体育设施，积极备战省第十三届运动会。继续开展1%人口抽样调查工作。大力发展妇女儿童、老龄、残疾人事业，继续做好外事侨务、新闻出版、档案方志等工作。

　　确保安全稳定。严格落实“党政同责、一岗双责、失职追责”的安全生产责任制，加强煤矿、非煤矿山、危险化学品、交通运输、建筑施工、特种设备、消防等重点行业领域和人员密集场所的安全监管，做好地质灾害防治、防汛抗旱和森林防火工作，推进“打非治违”常态化，抓好安全社区建设，提高科技兴安水平，严防重特大安全事故发生。实施最严格的食品药品监管，确保食品药品安全。深化“平安内江”建设，健全立体化社会治安防控体系，依法打击各类违法犯罪活动。完善社会稳定风险评估配套机制，实行重大涉信、涉稳问题挂牌化解。深化社区矫正，加强法律援助。开展信访“三无”（31）创建活动，创新“大调解”机制，规范信访秩序，确保“六个不发生”（32）。完善应急机制，加强应急指挥平台建设，提高应急处突能力。强化国防动员和后备力量建设，做好双拥共建、人民防空等工作。

　　（七）坚持依法行政、廉洁自律，着力加强自身建设

　　讲法治。把依法行政、厉行法治作为政府工作的基本准则，将政府工作全面纳入法治轨道，依法全面履行政府职能。坚持重大事项向人大报告、规范性文件向人大备案，主动接受人大工作监督、法律监督和政协民主监督，认真办理人大代表议案、建议批评意见和政协提案。加强与工会、共青团、妇联等群团组织的联系。严格遵循重大行政决策法定程序，建立健全决策咨询制度，加强新型智库建设，提升依法、科学、民主决策水平。推进综合执法，加强行政复议和行政应诉工作。推动行政权力平台有效运行，深化政务信息公开。加大普法工作力度，深入开展“法律七进”活动。

　　重实干。把崇尚实干、拒绝空谈作为政府工作的基本追求，以严的要求、实的作风推进各项工作。坚持少说多做，说了就算、定了就干，力戒“虚言”“伪事”，做到“抓铁有痕、踏石留印”。坚持紧抓快办，提升效能，力戒“懒政”“怠政”，做到“案无积卷、事不过夜”。坚持善做善成，以“钉钉子”精神，一级抓一级、层层抓落实，力戒“中梗阻、冷横推”，做到每项任务有部署、有检查、有效果。

　　敢担当。把敢于担当、忠诚履责作为政府工作的基本要求，做到日常工作能尽责、难题面前敢负责、出现过失勇担责。敢于“担急”，遇到紧急情况、自然灾害、突发事件，勇于站在一线，第一时间掌握情况，第一时间解决问题。敢于“担难”，勇于到发展慢、基础差的地方去研究方法，到问题多、意见大的地方去化解矛盾，到工作推不开、情况较复杂的地方去打开局面。敢于“担责”，善于运用辩证思维、底线思维、法治思维来处理问题、解决矛盾、推动工作，用脑用心、尽职尽责，既推崇创新、又宽容失败，推动更多工作走在全国全省前列。
